# Comparison of the Phenolic Compounds, Carotenoids and Tocochromanols Content in Wheat Grain under Organic and Mineral Fertilization Regimes

**DOI:** 10.3390/molecules171012341

**Published:** 2012-10-19

**Authors:** Iwona Konopka, Małgorzata Tańska, Alicja Faron, Arkadiusz Stępień, Katarzyna Wojtkowiak

**Affiliations:** 1 Department of Food Plant Chemistry and Processing, Faculty of Food Science, University of Warmia and Mazury in Olsztyn, Olsztyn 10-726, Poland; Email: m.tanska@uwm.edu.pl (M.T.); alicja.faron@uwm.edu.pl (A.F.); 2 Department of Agriculture Systems, Faculty of Environmental Management & Agriculture, University of Warmia and Mazury in Olsztyn, Olsztyn 10-727, Poland; Email: arkadiusz.stepien@uwm.edu.pl; 3 Department of Fundamentals of Safety, Faculty of Technical Sciences, University of Warmia and Mazury in Olsztyn, Olsztyn 10-266, Poland; Email: katarzyna.wojtkowiak@uwm.edu.pl

**Keywords:** wheat grain, meat bone meal, organic fertilization, phytochemicals

## Abstract

A field study was performed to evaluate the effect of mineral (NPK) and organic-based fertilizers such as compost (C), manure (FYM) and meat and bone meal (MBM) on the appearance (dimensions and color) of spring wheat kernels and on the total content in grain of main its phytochemicals (polyphenols, carotenoids and tocochromanols) and phenolic acids composition. Total phenolic compounds were determined using the Folin-Ciocalteu assay after alkaline hydrolysis of grain and carotenoids were analyzed spectrophotometrically. Composition of tocochromanols and phenolic acids was determined using RP-HPLC techniques. Only insignificant differences in the appearance of kernels and small changes in the content and composition of grain phytochemicals were noted between the studied fertilization systems. Among the analyzed phytochemicals the greatest variation was observed in the group of polyphenol compounds, with a stated increase of their total content of 6.7 and 11.2% in grain fertilized with MBM and compost, respectively. Simultaneously the grain from organic fertilization contained significantly less phenolic acids, and the decrease in their content ranged from 10.0% for FYM to 24.8% for MBM+EM-1. Organically and conventionally fertilized grain had similar amounts of tocochromanols and carotenoids. Comparison of MBM and MBM+EM-1 variants showed that application of effective microorganisms decreased carotenoids and tocochromanols content by 8.5 and 9.7%, respectively.

## 1. Introduction

Whole grain products are recommended as components of the human primary diet [1]. Apart from supplying a considerable amount of calories, proteins and carbohydrates, their daily consumption also provides a range of ingredients, cumulatively known as phytochemicals [2,3]. These are phytoactive, non-nutritional substances with diverse actions and a generally positive effects on human health. They act as free radicals scavengers, and possibly prevent the development of cancer, cardiovascular diseases, type II diabetes, obesity and inflammation [2,4]. The main phytochemicals found in wheat grain include polyphenols, carotenoids and tocochromanols [2,5,6].

The quality of wheat yield is the effect of the interaction of genetic (variety-related) and environment-related factors during plant vegetation. The type and amount of fertilizers used are regarded as being of decisive importance for the cereal yield and the content of fundamental chemical compounds. Wheat cultivation commonly involves use of nitrogen-phosphorus-potassium (NPK) fertilizers, whose purpose is to ensure the right ratio of N:P:K in the soil at about 150:60:110 kg·ha^−1^. A positive correlation between the amount of NPK fertilizers used and the yield of grain and its quality has been confirmed by many researchers [7,8,9].

Interest in organic foods is currently growing. Consumers find it more healthy and of better quality due to its lower content of pesticides and nitrates as well as higher nutritional value [10,11]. Organic farming of wheat involves using organic fertilizers. The most commonly applied include manure and compost. They provide organic matter to the soil, which is transformed into humus by earthworms and microorganisms [10,12]. Moreover, they contain easily available forms of nitrogen, phosphorus (P_2_O_5_) and potassium (K_2_O) in the amounts necessary for development and high yield of plants, and they also inhibit weed germination [12,13]. Organic fertilizers increase soil water potential and the amount of water available to plants. Thereby, they have a positive effect on the absorption of minerals during the periods of water shortage in soil, which results in increased yield, thousand kernels weight and protein concentration in grain [14,15].

Another source of elements occurring in artificial fertilizers are meat and bone meals (MBM). They contain about 8% N, 5% P, 1% K and 10% Ca [16]. Before 2,000 they were used as fodder additives; but, due to the risk of transmitting vectors of infectious diseases (TSE and BSE), they were banned in the EU countries. However, the EU allows the use of MBM as organic fertilizers (EC No. 181/2006). Therefore, use of MBM has an indirect and positive effect on the environment because it restricts the demand for artificial fertilizers, while at the same time enabling disposal of huge amounts of waste from the meat processing industry [16,17,18,19,20]. Absorption of minerals present in MBM can be improved by applying effective microorganisms (EM) which are able to carry out targeted processing of nutrients present in soil and to make it more fertile [21,22].

There have been many reports lately on the effect of organic fertilization on the content of the main plant storage polymers (carbohydrates and proteins) as well as micro- and macroelements [16,17,18]. However, there have been few reports comparing the effect of different forms of fertilization on the content of nutritionally valuable phytochemicals. Zuchowski *et al.* [11], Taie *et al.* [23], and Omar *et al.* [24] observed an increase in concentration and change of the composition of phenolic compounds in wheat grain, soy bean and cassava tubers, respectively. On the other hand, Hussain *et al.* [25] found that in organically grown wheat the content of tocochromanols was in a similar range as reported for conventionally grown wheat. Stracke *et al.* [26] concluded that climate factors have a greater impact on the carotenoids and phenolic acids concentrations in wheat grain than production methods (organic *vs*. conventional). 

The present study aimed at evaluating whether wheat grain produced conventionally and organically (with the use of compost, manure and meat and bone meal) differs in the content of total polyphenols and phenolic acids, carotenoids and tocochromanols.

## 2. Results and Discussion

### 2.1. Kernel Characteristics

**Figure 1:**
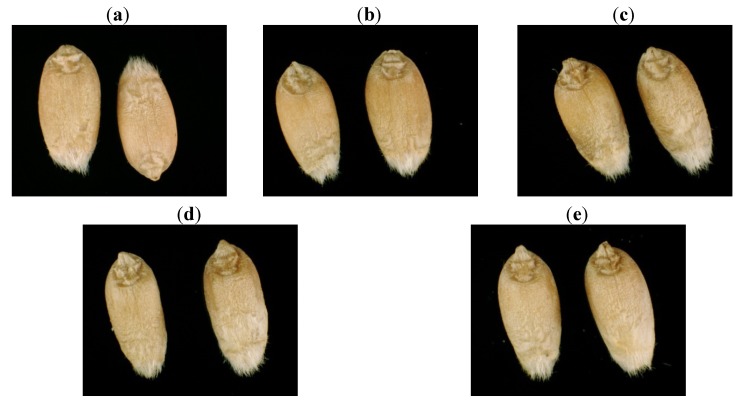
Images of wheat kernels from different systems of fertilization: (**a**) NPK, (**b**) compost, (**c**) FYM, (**d**) MBM, (**e**) MBM+EM-1.

Thousand kernels weight (TKW) ranged from 33.79 to 35.29 g (Table 1). These are typical values for spring wheat kernels, which are usually smaller than those of winter wheat [26,27,28,29]. It has been shown that only compost and MBM+EM−1 changed TKW of wheat grains in comparison to the NPK system. The kernels from plots fertilized with compost were 2.2% heavier, and kernels from plots fertilized with MBM+EM−1 of 2.1% lighter than traditionally fertilized ones. The average length and width of the kernels ranged from 7.15 to 7.30 mm and from 3.15 to 3.24 mm, respectively (Table 1). Although the differences relative to the NPK fertilization variant did not exceed 2%, compost fertilization resulted in slight, but significant shortening of kernels. The kernels were also wider than those obtained with the other variants of organic fertilization (Figure 1). This resulted in the lowest value of the length/width ratio (2.21). On the contrary the highest value of that index was determined for the sample fertilized with MBM+EM−1. The fertilization variants caused only slight differences in the color of the kernel surface (Table 1). The most varied values of the color components were observed for kernels fertilized with NPK (H = 27.23, S = 28.01, I = 60.13) and those treated with FYM (H = 28.02, S = 30.76, I = 57.70). This suggests that the kernels from conventional cultivation were slightly lighter and more red than others.

**Table 1:** Mass, geometrical features and color of wheat kernels from different systems of fertilization.

System of fertilization	NPK		C		FYM		MBM		MBM+EM-1	
	mean	range	mean	range	mean	range	mean	range	mean	range
1000 kernels weight (g)	34.52 ± 0.47a	34.01–35.13	35.29 ± 0.39b	34.63–35.64	33.85 ± 0.68ac	32.77–34.58	33.83 ± 0.55ac	33.24–34.48	33.79 ± 0.42c	33.16–34.18
Kernel length (mm)	7.26 ± 0.39a	5.59–8.19	7.15 ± 0.4b	5.97–8.43	7.22 ± 0.39ab	6.05–8.19	7.30 ± 0.37a	5.97–8.14	7.26 ± 0.42a	5.99–8.54
Kernel width (mm)	3.20 ± 0.24ab	2.48–3.68	3.24 ± 0.26b	2.51–3.86	3.15 ± 0.26a	2.23–3.90	3.18 ± 0.26a	2.36–3.83	3.15 ± 0.25a	2.18–3.75
Length/width ratio (-)	2.27 ± 0.15a	1.95–2.78	2.21 ± 0.42b	4.76–7.09	2.30 ± 0.17ac	1.88–2.98	2.30 ± 0.16a	1.92–2.87	2.32 ± 0.18c	1.91–2.94
Kernel surface H (°)	27.23 ± 0.95a	23–28	27.90 ± 0.52b	26–30	28.02 ± 2.12b	25–31	27.93 ± 0.39b	26–30	27.63 ± 0.96c	22–30
Kernel surface S (%)	28.01 ± 3.42a	12.5–35.55	30.52 ± 3.39b	23.05–42.19	30.76 ± 3.64b	23.83–51.56	29.64 ± 3.34c	18.36–37.89	28.6 ± 3.38a	18.36–41.41
Kernel surface I (%)	60.13 ± 3.06a	52.73–74.74	58.24 ± 2.74bc	49.22–63.8	57.7 ± 3.2b	37.37–63.67	58.57 ± 2.75cd	51.69–67.06	59.13 ± 3.08d	50.91–69.01

Within each line, means with the same letter are not significantly different (*p* < 0.05).

### 2.2. Chemical Composition

The results of determination of free and total phenolic compounds are presented in Table 2. 

**Table 2:** Content of phenolic acids (FA) and free and total phenolic compounds in wheat grain from different systems of fertilization (μg/g of seed dry mass).

Compound *	System of fertilization				
	NPK	C	FYM	MBM	MBM+EM-1
	**Phenolic compounds composition**				
Free phenolics	449.6 ± 14.5a	509.0 ± 61.2ab	506.8 ± 95.4ab	515.6 ± 20.7ab	530.2 ± 12.4b
Total phenolics – EE	1249 ± 41.6a	1277 ± 77.9ab	1328 ± 33.0ab	1282 ± 119.5ab	1363 ± 32.7b
Total phenolics – ME	1460 ± 143.5ab	1739 ± 124.3c	1487 ± 14.8ab	1608 ± 122.9bc	1414 ± 125.0a
Total phenolics	2710 ± 101.9a	3016 ± 202.3b	2816 ± 47.8ac	2891 ± 3.3bc	2777 ± 157.7ac
Free/total phenolics	16.60	16.88	18.00	17.83	19.09
	**Phenolic acids**				
ferulic	538.8 ± 31.1a	440.7 ± 35.0bc	479.2 ± 51.6b	420.5 ± 10.8c	418.3 ± 4.2c
*p*-coumaric	45.4 ± 2.5a	32.6 ± 2.5b	37.3 ± 0.3c	38.4 ± 1.6c	19.8 ± 0.2d
sinapic	86.1 ± 19.0a	77.8 ± 10.7ab	88.6 ± 7.1a	86.6 ± 15.8a	68.3 ± 3.4b
vanillic	10.8 ± 0.1a	9.0 ± 0.1b	8.9 ± 0.6b	9.3 ± 0.8b	7.5 ± 0.3c
*p*-OH benzoic	5.7 ± 0.2a	4.4 ± 0.1b	4.4 ± 0.3b	4.7 ± 0.0c	3.5 ± 0.0d
sum of phenolic acids	686.8 ± 14.7a	564.6 ± 48.5c	618.4 ± 59.9b	559.5 ± 4.2c	517.3 ± 1.4c

* soluble in 80% methanol (free phenolics) and in diethyl ether (EE) and in 80% methanol (ME) after alkaline hydrolysis (total phenolics); Within each line, means with the same letter are not significantly different (*p* < 0.05).

The total content of polyphenols ranged from 2,710 to 3,016 µg/g of grain. The grains from NPK fertilization contained the lowest concentration of these compounds, and the highest value was seen in grain which was fertilized with compost. The ether-soluble fractions accounted for 42% to 49% of them. Only a small part of the total polyphenols occurred as soluble forms extracted by 80% methanol. The lowest concentration was found in the sample of grain fertilized with NPK (16.6%) and the highest was in the sample fertilized with MBM+EM−1 (19.1%).

An analysis of the phenolic acids composition has shown that the wheat grain contained the following acids: ferulic, sinapic, *p*-coumaric, vanillic and *p*-OH benzoic (Table 2). The dominant ferulic acid accounted for 75% to 81% of the total content, and the proportions of the other acids were as follows: sinapic acid—from 12% to 15%, *p*-coumaric acid—from 4% to 7% and both of vanillic and *p*-OH benzoic acids—approx. 2% of the total content. All the samples from organic farming contained significantly less phenolic acids as compared with the grain treated with NPK. It was the most visible in grain from MBM−EM−1 cultivation system that contained about 1/3 less total phenolic acids than the grain from NPK fertilization.

The content of free and total carotenoids is presented in Table 3. Depending on the fertilization systems, the wheat grain contained from 1.38 to 1.54 μg/g of petroleum ether soluble pigments and from 3.54 to 3.87 μg/g of total yellow pigments. Generally, the differences between the samples were small, but the grain treated with MBM was found to contain the largest amounts of carotenoids. Supporting the fertilization with effective microorganisms reduced the accumulation of carotenoids in wheat grain. The ether-soluble fractions accounted for 36.4% to 42.1% of total carotenoids, and was the highest in NPK fertilized grain. This suggests that more polar than carotene xantophylls predominated in the tested grain. 

**Table 3:** Content of carotenoids (CAR), tocopherols (T) and tocotrienols (T3) in wheat grain from different systems of fertilization (μg/g of seed dry mass).

Compound *	System of fertilization				
	NPK	C	FYM	MBM	MBM+EM−1
	Carotenoids				
Soluble CAR	1.54 ± 0.01a	1.51 ± 0.18ab	1.51 ± 0.15ab	1.41 ± 0.01ab	1.38 ± 0.01 b
Total CAR	3.60 ± 0.03a	3.59 ± 0.10a	3.77 ± 0.12b	3.87 ± 0.20b	3.54 ± 0.09 a
	Tocochromanols				
Soluble α-T	13.49 ± 0.63a	14.19 ± 0.38b	14.02 ± 0.53ab	15.09 ± 0.10c	13.40 ± 0.39a
Total α-T	14.61 ± 0.45a	15.20 ± 0.05a	14.93 ± 0.24a	15.48 ± 1.10a	13.56 ± 0.84b
Soluble β-T	3.51 ± 0.09a	3.58 ± 0.03a	3.60 ± 0.08a	3.53 ± 0.07a	3.08 ± 0.11b
Total β-T	5.73 ± 0.25a	5.89 ± 0.16a	6.10 ± 0.07a	5.86 ± 0.14a	5.15 ± 0.24b
Soluble α-T3	5.52 ± 0.10a	5.50 ± 0.13ab	5.63 ± 0.12b	5.34 ± 0.26b	5.16 ± 0.11c
Total α-T3	6.29 ± 0.48a	5.98 ± 0.22a	5.43 ± 0.12b	6.08 ± 0.04a	5.38 ± 0.30b
Soluble β-T3	17.95 ± 0.29ab	18.30 ± 0.62bc	17.76 ± 0.03ab	18.56 ± 0.42c	17.44 ± 0.51a
Total β-T3	18.86 ± 0.24ab	19.14 ± 0.18a	18.75 ± 0.38bc	18.69 ± 0.33c	17.55 ± 0.04d
Soluble T	17.00 ± 0.53a	17.77 ± 0.35b	17.62 ± 0.60b	18.62 ± 0.17c	16.48 ± 0.50a
Total T	20.34 ± 0.54a	21.09 ± 0.18ab	21.03 ± 0.12ab	21.34 ± 1.35b	18.71 ± 0.95c
Soluble T3	23.47 ± 0.77a	23.80 ± 0.87a	23.39 ± 0.08a	23.90 ± 0.32a	22.60 ± 0.87b
Total T3	25.15 ± 0.02a	25.12 ± 0.34a	24.18 ± 0.31b	24.77 ± 0.47ab	22.93 ± 0.19c
Soluble T+T3	40.47 ± 1.30a	41.57 ± 1.22ab	41.01 ± 0.68ab	42.52 ± 0.48b	39.08 ± 1.38c
Total T+T3	45.49 ± 0.53a	46.21 ± 0.52a	45.21 ± 0.43a	46.11 ± 1.82a	41.64 ± 1.15b
Soluble T3/T	1.38 ± 0.08a	1.34 ± 0.03ab	1.33 ± 0.04ab	1.28 ± 0.05b	1.37 ± 0.03a
Total T3/T	1.24 ± 0.03a	1.19 ± 0.02ab	1.15 ± 0.03b	1.16 ± 0.06b	1.23 ± 0.05a

* extractable by petroleum ether (soluble) and by water-saturated butanol (total); Within each line, means with the same letter are not significantly different (*p* < 0.05).

The wheat samples contained both forms of tocochromanols (Table 3). Approximately 90%–94% of them were extractable by petroleum ether. Analysis of (total) water-butanol soluble tocochromanols showed that α- and β-tocopherol ranged from 13.56 to 15.48 μg/g and from 5.15 to 6.10 μg/g, respectively. Their total content was the highest in the grain fertilized with MBM (21.34 µg/g), but use of EM−1 removed that positive effect. The tocotrienol fraction was found to contain two main forms: β-T3 (from 17.55 to 19.14 μg/g), α-T3 (from 5.38 to 6.29 μg/g) and traces of γ-T3 (below 0.4 μg/g—data not presented).

The total tocotrienol content ranged from 22.93 to 25.15 μg/g. The combined content of both forms of tocochromanols was the highest in the C (compost) and MBM samples, but they were not statistically different from the NPK variant. Using effective microorganisms reduced the total content of tocochromanols by as much as 8%, to a lower level than that determined for the NPK variant. The ratio of both forms in water-butanol extracts ranged from 1.15 to 1.24, with tocotrienols dominating.

### 2.3. Discussion

The size, shape, weight and color of kernels determine the commercial value of wheat grain. These features are associated with its technological and milling quality and the baking value of flour. In general, big grains containing large amounts of nutrients, with uniform color and dimensions, are more desirable for food processing. In hexaploid wheat, these traits are controlled by a range of quantitative traits loci (QTL) on chromosomes of all the three genomes A, B and D [27,28]. However, it has been well-documented that the environmental conditions can also change the cultivar characteristics. For example, high temperatures and a water deficit significantly reduce kernel weight and dimensions (thickness and width) [29,30].

Of the phytochemicals under investigation, polyphenols are a more diverse group, both in the qualitative and quantitative aspect. Their content lies within a wide range from about 800 to 2,400 µg/g of dry matter of grain [3,31,32]. They include phenolic acids at up to 700 µg/g [11], flavonoids at up to 500 µg/g [32,33], condensed tannins at up to 700 µg/g [34], alkylresorcinols at up to 800 µg/g [35,36,37] and lignans at up to 4 µg/g [33,38]. The results of our study indicate that the total content of phenolic compounds is equal to 2,700–3,000 µg/g of dry matter, which is slightly higher than in the papers cited above. This may be explained by the effect of alkaline hydrolysis of grain, that releases phenol aglycons from ester and glycoside compounds with cell-wall polysaccharides [39,40] and decomposes of other grain components, which can subsequently react with the Folin-Ciocalteu (F-C) reagent [41]. Everette *et al.* [41] even suggest that F-C assay should be rather seen as a method to measure total antioxidant capacity than phenolic content. From this point of view the values of approximately 3,000 µg/g indicate high antioxidative potential of the grain samples under investigation, which was the highest in the sample fertilized with compost and the lowest in the sample treated with NPK. Phenolic acids were responsible only for approx. 20%–25% of the potential. The main phenolic acid was ferulic acid, that may account for as much as 90% of total phenolic acids [3,11].

Although there have been many reports on polyphenols in cereal grain, data on the effect of cultivation/fertilization on their content are scarce. Most of them indicate that phenolic compounds content is higher in grain from organic cultivation. According to Zuchowski *et al.* [11] the observed higher concentration of phenolic acids in organic wheat is caused mainly by the smaller kernel size. Taie *et al.* [23] explained this phenomenon by the action of bioorganic fertilizers that help plants to fix nitrogen from the air and utilize it to production phytohormones and other growth-promoting compounds [42], such as phenolics. Our results confirmed the higher accumulation of total polyphenols in grain from organic cultivation, although those kernels contained significantly less phenolic acids (on average of 18%).

One of the factors which affects the biological activity of phenolic compounds is their bioavailability from the diet. First of all, the form in which they enter alimentary tract (free or bound) is important. Monomers and dimers which are part of the free fraction are more easily available and they are imbibe in the higher sections of the alimentary tract [43,44]. From this point of view, grain produced by organic cultivation is more valuable because it contains more free polyphenols, with the largest increase observed for MBM+EM-1 fertilization. This may have been affected by enzymatic activity of grain microflora. Suproniene *et al.* [45] have shown that increasing the amount of mineral fertilizers favors infection of the surface of spring wheat grain by *Fusarium* and *Penicillium* species. These fungi produce esterases, which are part of the enzymatic spectrum employed to degrade plant polysaccharides and to release phenolic compounds [46,47,48].

Moreover, the amount and proportions of tocochromanols in wheat grain samples vary and lie within a wide range from 10.2 to 74.3 μg/g for the content, and from 1.2 to 5.3 for the tocotrienol/tocopherol ratio [3,25,31,49,50,51]. The results of this study lie in the middle of the range for the content and at the lower boundary of the range for the ratio. The reports on the effect of fertilization for this group of compounds have also been scarce. According to Hussain *et al.* [25] organically grown wheat contains similar amounts of tocochromanols to that found in conventionally grown wheat (a study conducted in Sweden). These conclusions have been confirmed by our results, because the differences relative to the NPK sample did not exceed 5%. Synthesis of tocochromanols in plastids and chloroplasts is affected by stress and/or senescence of grain [52,53,54]. The main function of tocochromanols is to protect PUFA in cellular membranes against oxidative stress; they also regulate the process of adaptation to cold and the germination process [53,54]. Studies conducted for several crop species have shown that synthesis of α-tocopherol is favored by such factors as drought, heat, salinity and UV/light [55,56]. This is probably one of the reasons for the large variability in tocochromanols content in wheat grain which have been reported in the literature. Another reason is the different extraction techniques, which was mentioned by Okarter *et al.* [3].

Wheat grain usually contains 1.3 to 5.0 µg/g of carotenoids [3,34,57]. They are deposited throughout the whole kernel in different wheat species. The main compound of these pigments is lutein, whose total content ranges from about 50% to 95% [3,58,59]. The present results found the content of carotenoids to be within the cited range. We can state that organic fertilizers only slightly affected accumulation of these compounds (differences not exceed 7%). Similar results were previously obtained by Stracke *et al.* [26] who stated that climate has a greater impact on the carotenoids concentration than the production method. Organic fertilization have also slightly changed the ratio of ether-soluble and water-butanol-soluble fractions of carotenoids. Generally, the lowest content of less polar forms (ether-soluble) has been found in grain fertilized with MBM (alone and with addition of effective microorganisms). This may possibly affect antioxidative function of carotenoids in grain-based diet.

## 3. Experimental

### 3.1. Plot Experiments

Samples of spring wheat grain of the Tybalt cultivar, cultivated in 2009 in the experimental field in Bałcyny in the north-eastern part of Poland (53°36'N, 19°51'E) were used as the study material. A single-factorial field study was carried out. The experiment was set up in a random block design, with four replicates. The experimental design covered five systems of fertilization (Table 4). 

Effective microorganisms were used only on the plot fertilized with meat and bone meal at 5 dm^3^·ha^−1^, in two doses: (a) 3 dm^3^·ha^−1^—immediately before sowing, (b) 2 dm^3^·ha^−1^—before the first weeding procedure. The EM-1 preparation made by the Greenland Technologia EM Company (Trzcianki, Poland) was used. Microorganisms were revived by adding 1 dm^3^ of EM−1 with 4 dm^3^ of water sweetened with 40 g of molasses and leaving it for 12 h at the temperature of 20 ± 2 °C. The preparation was water-diluted and used as a spray in the dose of 300 dm^3^·ha^−1^. Spraying was done on humid and cloudy days, immediately before mechanical agricultural procedures. The composition of the microflora in used preparation was not determined, but Szymański and Patterson [60] and Valarini *et al.* [61] have previously reported that it contains bacteria—*Lactobacillus plantarum*, *L. casei*, *Streptococus lactis*, *Rhodopseudomonas palustrus*, *Rhodobacter spae*; yeast—*Saccharomyces albus*, *Candida utilis*; Actinobacteria—*Streptomyces albus*, *S. griseus* and fungi—*Aspergillus oryzae*, *Mucor hiemalis*.

**Table 4:** Doses of nutrients in kg·ha^−1^, with fertilizers.

Content of mineral compound	NPK	Compost	Farm Yard Manure	Meat and Bone Meal	Meat and Bone Meal + Effective Microorganisms (MBM+EM−1)
[kg·ha^−1^]		(C)	(FYM)	(MBM)	
N	90.0	71.0	51.0	99.8	99.8
P	31.0	29.0	12.1	59.7	59.7
K	83.0	62.0	49.0	6.2	6.2
Mg	-	16.0	8.0	3.0	3.0
Ca	-	52.0	34.0	28.5	28.5
Na	-	3.8	3.2	8.4	8.4
Cu	-	0.048	0.050	0.015	0.015
Fe	-	5.58	3.85	0.77	0.77
Mn	-	0.740	0.450	0.005	0.005
Zn	-	0.504	0.250	0.149	0.149
Dose of fertilizer		10 t·ha^−1^	10 t·ha^−1^	1.5 t·ha^−1^	1.5 t·ha^−1^

### 3.2. Grain Features Measurement

Harvested grain was dried to a moisture content below 15%. It was then cleaned of dust and broken kernels on a laboratory air-sieve separator (fragments with width/thickness below 2.2 mm were rejected) and stored at a temperature of 6 ± 2 °C. The weight of 1,000 kernels (TKW) was analyzed using an LN-S-50 seed counter in five replicates. The kernel dimensions: length, width and length/width ratio (elongation) and color of surface were determined for 60 kernels using the digital image analysis according to Konopka *et al.* [57]. The images were acquired by a high resolution, low-noise CCD Nikon DXM-1200 color camera and analyzed by LUCIA G ver. 4.8 software. The results are presented in HSI (H-hue, S-saturation, I-intensity) color space, where H is expressed in degrees, and S and I in percentages.

### 3.3. Extraction of Grain Phytochemicals

**Figure 2:**
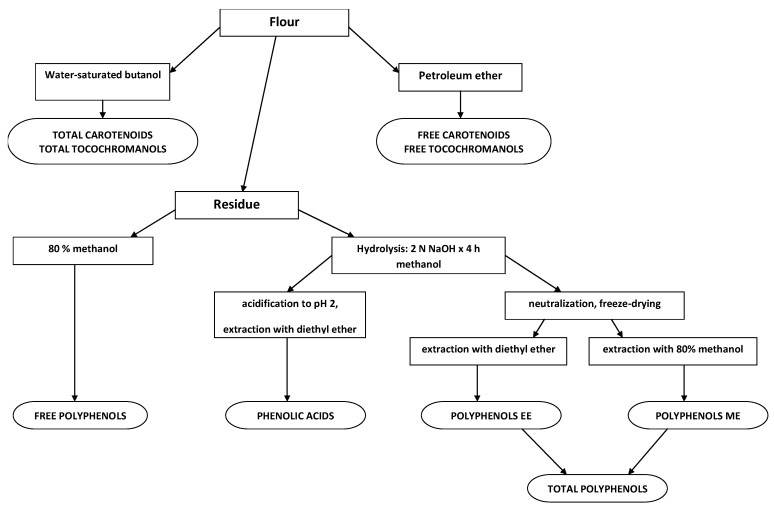
Scheme of carotenoids, tocochromanols and polyphenols extractions.

Before the chemical analyses, the grain was ground to obtain particles smaller than 300 µm. The carotenoids, tocochromanols and polyphenols extraction scheme is presented on Figure 2. Free lipophilic compounds (carotenoids, tocochromanols) were extracted without a saponification step by petroleum ether and free polyphenols by 80% methanol. Total carotenoids and tocochromanols were extracted by water-saturated butanol. Extraction of total polyphenols was preceded by alkaline hydrolysis of wheat samples with 2 N NaOH × 4 h at room temperature. After hydrolysis, the mixture was neutralized (2 N HCl) and evaporated to dryness. Released polyphenols were extracted in two steps (1) by the use of diethyl ether and (2) by the use of 80% methanol. Additionally, the composition of phenolic acids was determined in the extract of total polyphenols according to method of Robbins [62]. To prevent phytochemicals oxidation the extraction procedures were done in the presence of a mixture of butylated hydroxytoluene (BHT). Extractions with petroleum ether were conducted in a FoodALYT RT 60-type Soxhlet apparatus (Omnilab). Extractions with water-saturated butanol were conducted by the method proposed by Kaneko [63], and that of polyphenols using the modified method proposed by Irmak *et al.* [64]. All the extracts were concentrated to dryness at temperatures below 50 °C. 

### 3.4. Determination of Total Carotenoids Content

Carotenoids content was determined spectrophotometrically by the method described by Craft [65]. To this end, 2.5% extract solutions in hexane were prepared and their absorbance was measured at the wavelength of 454 nm (maximum of lutein absorption). The measurements were carried out with a UNICAM UV/Vis UV2 spectrophotometer. Carotenoid content was calculated based on molar absorptivity coefficient (for lutein dissolved in hexane is equal to 147,300 L/mol·cm) and molar mass of lutein (equal to 568.87 g/mol) [65]. The results are presented as μg/g of a sample dry mass.

### 3.5. Determination of Tocopherols and Tocotrienols Content

The tocopherols and tocotrienols content was determined by the RP-HPLC technique using the method described by Gimeno *et al.* [66]. One % solutions of ether extract and butanol-water extract in hexane were prepared. After being stirred and centrifuged in a 5417R type Eppendorf centrifuge (10 min, 25,000 × *g*), they were transferred to chromatography vials. The analysis was carried out in a series 1200 Agilent Technologies apparatus, fitted out with a fluorescence detector of the same manufacturer. Separation was done on a LiChrospher Si 60 column (5 μm, 250 mm × 4 mm) manufactured by Merck, with 0.7% isopropanol solution in hexane as the mobile phase. Identification of the isomers was based on retention times, determined for reference standards of tocopherols and tocotrienols (Sigma-Aldrich). The content of each isomer was determined from calibration curves of the reference standards and expressed as μg/g of a sample dry mass.

### 3.6. Determination of Total and Free Polyphenols Content

The content of phenolic compounds (free and total) was determined spectrophotometrically with Folin-Ciocalteau reagent by the method described by Ribereau-Gayon [67]. The color reaction was carried out by adding Folin-Ciocalteau reagent (0.5 mL), 14% sodium carbonate (3 mL) and distilled water (6.5 mL) to the polyphenols extract. After mixing, the solutions were left for 60 min and their absorbance was then measured against the reagent sample (without the phenolics extract) at the wavelength of 720 nm, with a UNICAM UV/Vis UV2 spectrophotometer. The content of phenolic compounds was expressed as μg of D-catechin equivalent in 1 g of a sample dry mass.

### 3.7. Determination of Phenolic Acids Content

The phenolic acid content was determined by the RP-HPLC technique using the method described by Ogrodowska *et al.* [68]. Phenolic acids were extracted with ethyl ether from flour sample hydrolysates (hydrolysis as for total phenols), acidified to pH 2. Then the ether was evaporated, and the dry residue was dissolved in methanol, centrifuged in a 5417R-type Eppendorf centrifuge (10 min, 25,000 × *g*) and transferred to chromatography vials. Liquid chromatography (RP-HPLC) was performed on an Agilent Technologies 1200 series system fitted with a photodiode detector and with a Phenomenex Synergi Fusion RP18 column (4 μm, 2 mm, 150 mm) at the temperature of 30 °C. The mobile phase consisted of two solvents: A—0.15% formic acid (FA) in acetonitrile and B—0.15% FA in water. The gradient applied was: 0–7 min 10% of eluant A, followed by linear increase up to 100% of eluant A over 43 min. The flow rate was equal to 0.2 mL/min. Detection was performed at the wavelength of 280 and 320 nm. Phenolic acids were identified by comparing with absorption spectra of the reference phenolic acids. The content of phenolic acids was determined from calibration curves of phenolic acid reference standards and expressed as μg of D-catechin equivalent in 1 g of a sample dry mass.

### 3.8. Statistical Analysis

All the chemical determinations were performed in triplicate. The experimental results were analyzed using Statistica 8.0 software. ANOVA analysis with Duncan tests was performed at the significance level of *p* < 0.05.

## 4. Conclusions

The data suggest that despite the use of diverse fertilization systems, wheat grain did not vary much, either in terms of the content of the phytochemicals under investigation or the morphological features of the grain. Organically and conventionally fertilized grain had similar amounts of tocochromanols and carotenoids. However, organic wheat grain was more abundant in total polyphenols, while less abundant in phenolic acids. Only minor variations were found in the content of phytochemicals among the tested organic fertilizers. Applied effective microorganisms had a negative influence because they contributed to a decrease of tocochromanols, carotenoids and phenolic acids content in the grain, along with a decrease in kernel weight.
